# Development of a rabbit corneal equivalent using an acellular corneal matrix of a porcine substrate

**Published:** 2008-11-30

**Authors:** Yong-gen Xu, Yong-sheng Xu, Chen Huang, Yun Feng, Ying Li, Wei Wang

**Affiliations:** Department of Ophthalmology, Peking University Third Hospital, Beijing, China

## Abstract

**Purpose:**

The tissue equivalent that mimics the structure and function of normal tissue is a major bioengineering challenge. Tissue engineered replacement of diseased or damaged tissue has become a reality for some types of tissue such as skin and cartilage. The tissue engineered corneal epithelium, stroma, and endothelium scaffold are promising concepts in overcoming the current limitations of a cornea replacement with an allograft.

**Methods:**

The acellular corneal matrix from porcine (ACMP) was examined as a potential corneal cell sheet frame. The physical and mechanical properties of strength, expansion, transparency, and water content of the ACMP were measured. The major antigens of the cell components were completely removed with series of extraction methods, the major antigens of the cell components were identified by hematoxylin and eosin (HE), immunofluorescence staining, and scanning electron microscopy. The structural properties were investigated by HE stain and scanning electron microscopy. The three types of rabbit corneal cells were cultured in vitro, and characteristics were investigated by colony formation efficiency (CFE), BrdU staining, immunofluorescence staining, and western blot assay of keratin 3 (K3), vimentin, and aquaporin A. The biocompatibility of the ACMP was investigated for one month using rabbit corneal stroma and three types of cultured corneal cells both in vivo and in vitro. The three types of cultured rabbit corneal cells were seeded onto ACMP of each side at a cell density of 5.0×10^3^ cells/mm^2^.

**Results:**

The optical and mechanical properties of the ACMP were similar to the normal porcine cornea. The collagen fiber interconnected to the network, formed regular collagen bundles of the ACMP, and was parallel to the corneal surface. The ACMP was transferred to the rabbit cornea stroma, which showed an intact epithelium and keratocytes in the implant region. There were no inflamed cells or new vessel invasion one month after transplantation. The three types of cultured rabbit corneal cells were positive for K3, vimentin, and aquaporin A. CFE and BrdU (5-bromo-2′-deoxyuridine) staining showed no statistical difference. The cultured rabbit corneal limbal epithelial cells, keratocyte cells, and endothelial cells formed a confluent cell sheet on the ACMP, which consisted of one to two cell layers. Immunofluorescence and scanning electron microscopy examination showed that the cells steadily adhered to the surface of the ACMP and maintained their conformation and special molecule expression such as K3, vimentin, and aquaporin A. Rabbit corneal epithelium-ACMP, keratocytes-ACMP, and endothelium-ACMP scaffold was built in vitro.

**Conclusions:**

The rabbit corneal scaffold was made by the ACMP as a frame with three types of allogeneic rabbit corneal cells. This is a new concept in treating injured corneas.

## Introduction

The cornea is an avascular tissue that comprises one-sixth of the anterior surface of the eye and provides 75% of the refractive power needed for focusing light onto the retina. Injury to the cornea can lead to corneal opacification, visual impairment, and even blindness. At present, the only therapeutic option for dealing with such injury is corneal transplantation. This is the most successful tissue transplantation procedure in humans [1,2]. However, one limitation of this is its dependence on corneal availability from healthy donors. Recently, the situation has been aggravated in most regions of the world because the supply of donor tissue barely meets the ever-increasing demand. Therefore, an attractive alternative for dealing with the allograft shortage is to develop a corneal equivalent to restore the corneal function necessary for normal vision [3].

Xenografts promise sufficient resources of organs for clinical transplantation. The absence of blood vessels and the intracorneal production of immunosuppressive factors appear to allow corneal allografts to survive. However, the characteristic of “immunological privilege,” which makes the immune system ignorant of the presence of a graft, is not absolute in the xenograft. Evidence suggests that corneal xenografts ordinarily suffer severe rejection that is mostly mediated by reactive T-cells and antigen-presenting cells [4-6]. Recently, tissue engineering of the cornea has been presented as a promising concept in overcoming the limitations of corneal replacement with allografts. The principle of this type of tissue engineering is to create new, functional autologous tissues, which have the potential for regeneration and growth. The choice of an appropriate matrix for constructing tissue engineered cornea is crucial [7]. The ideal matrix should fulfill several requirements. It should be biocompatible and should allow for epithelization and repopulation with autologous recipient interstitial cells. Various types of matrices such as polymer or fibrin-gel scaffolds have been investigated [8]. Compared with these matrices, the porcine cornea appears particularly attractive because of its anatomic similarity to the human cornea [9]. Cell components of the cornea are the source of the antigens of the major histocompatibility complex responsible for allograft/xenograft rejection in various tissues. One way to overcome this might be the transplantation of corneal substrates without their cellular components. The special immunological or unspecific inflammatory response of the xenogeneic matrix is thought to be reduced by the decellularization procedure, and the matrix might subsequently be repopulated with recipient cells after implantation [10].

Recently, various decellularization procedures have been used to eliminate cells and create a cell-free matrix [8,11]. However, from these data, we conclude that the different decellularization procedures vary considerably in their efficacy so we have developed a protocol for this. In preclinical studies, porcine cornea is regularly used as an animal model due to its relative similarity to the human cornea [3] and its availability in great numbers from slaughterhouses. For this reason, various physical properties of the porcine cornea have been investigated using calorimetry, turbidimetry, tensile tests, and hydrothermal isometric tension measurements. Corneas of pigs, mice, rabbits, sheep, cats, dogs, and cows were quantitatively analyzed for water content, hydroxyproline, nucleic acid, total sulfated polyanion, chondroitin sulfate/dermatan sulfate, and keratan sulfate. Several samples or pools of tissue from each species were used. Water (% of wet weight), hydroxyproline (mg/g dry weight), and chondroitin sulfate (mg/g of hydroxyproline) contents were approximately constant across the species except for mice. The keratan sulfate content (mg/g of hydroxyproline) increased with corneal thickness whereas the dermatan sulfate content decreased. A simple model of mammalian corneal stroma has been tested against biochemical and ultrastructural measurements performed on several species. Water content, collagen, and total sulfated polyanion were constant. The biological corneal tissue reconstructed in vitro possessed three layers, the epithelium, the scaffold, and the endothelium. Xenogeneic corneal acellular matrix provides an ideal surface for corneal epithelial and endothelial cells’ adhesion and proliferation. Therefore, it is desired to be used as a scaffold for the reconstruction of the cornea in vitro [3,12,13].

Three different serial digestion methods were used to produce the acellular corneal stroma material. The biocompatibility of the materials was investigated by cell seeding, and the materials were implanted into the rabbit corneal stroma layer. The three cell types in the material were completely decellularized, and the collagen or elastic fibers were reserved integrally, showing a typical three-dimensional network. Rabbit corneal fibroblasts could expand on the materials in vitro. No obvious rejection could be observed, and the materials were gradually absorbed. Therefore, there was decellularization of the porcine cornea. Moreover, the rejection of the transplanted cornea is a common finding, and the key factor driving this immune rejection is known to be corneal endothelial damage. The results regarding the successful application of corneal allotransplantation suggest that the construction of the tissue engineered cornea with allogeneic epithelial cells, keratocytes, and endothelial cells has therapeutic potential. Thus, in this study, an active corneal epithelial, stromal, and endothelial equivalent has been reconstructed using porcine decellularized stroma components. The xenogeneic epithelial cells, keratocytes, and endothelial cells were then evaluated [13].

## Methods

### Animals

Fifteen New Zealand white rabbits (Peking University Health Science Center, Beijing, China) weighing 1.5–2.0 kg were used in this study. This research was performed under the guidelines of ARVO (Association for Research in Vision and Ophthalmology) and their statements for the use of animals in ophthalmic and vision research guides [14].

### Acellular porcine corneal matrix

Fresh porcine corneas were obtained from a local slaughterhouse. First, the corneal epithelium and endothelium were removed. This was done using 1.2 U/ml dispase II (Roche Applied Science, Penzbeg, Germany) at 4 °C for 16 h. The stroma was then trimmed to a 10 mm diameter section. To remove the hereditary material, the stromal discs were soaked in 1 mM Tris-HCl for 12 h, treated with a 1% Triton X-100 solution (Sigma, St. Louis, MO) at 4 °C for 12 h, digested with 0.25% trypsin-EDTA (Gibco-BRL Life Technologies, Gibco, Carlsbad, CA) at 37 °C for 30 min, and treated with DNase (Sigma) and RNase (Sigma) at 4 °C for 16 h. Between the treatment steps, the discs were washed twice with 5 mM Tris-HCl for 10 min. Finally, the scaffold materials were freeze-dried at −20 °C for 8 h, 0 °C for 8 h, and 20 °C for 4 h and then sterilized with gamma irradiation [15-18].

### Physical and mechanical characterizations of porcine acellular corneal matrix

#### Optical property

The primary acellular corneal matrix of porcine (ACMP) was placed on 35 mm culture dishes, and glycerol (90%) was added. They were dehydrated for 2 h and 4 h at room temperature. The light transparence and absorption were measured using a spectral photometer (DR5000; Hach, Loveland, CA). The primary cornea and the dehydrated ACMP were measured at 2 h and 4 h and compared to normal porcine cornea [3,19].

#### Mechanical properties

After sterilization by gamma irradiation, the dehydrated ACMP was tested strength, expansion, and water content. The rate of strength was investigated using a precise chest-developer (BAT1000; Aikoh, Tokyo, Japan) to elongate the dehydrated ACMP. The initial length (L1) and area (A1) of the normal porcine cornea and primary ACMP and the final length (L2) and area (A2) of the normal porcine cornea and the dehydrated ACMP were measured. The rate of strength was measured using the following formula:

(L2−L1)/(A2−A1)

The rate of water content was investigated using an electronic scale (AG135; Mettler Toledo, Schwerzenbanch, Switzerland) by the following formula:

(G2-G1)/G2 × 100%

where G1 is the weight of the primary condition of ACMP or the normal porcine cornea and G2 is the weight after being dried for 2 h at 65 °C.

The rate of expansion was measured using a counting cup (VC305; Branluebbe, **Norderstedt,** Germany) with the following equation:

(V1-V)/V1 × 100%

where V is the volume after dehydration for 2 h at 65 °C, V1 is the volume of the primary condition of the normal porcine cornea and ACMP. The overall experimental sizes of the specimens were 1 cm×1 cm [3,20,21].

### Cell culture

The New Zealand white rabbits were anesthetized with an intramuscular injection of 30 mg/kg of ketamine (Huawei, Shanghai. China) and 5 mg/kg of xylazine (Huawei). Local anesthetic was added with 0.5% proparacaine hydrochloride (Alcon, Fort Worth, TX). The limbal explants were dissected from the limbal zone, and the stromal explant was dissected from the stromal layer. Their sizes were 2.0 mm in width, 2.0 mm in length, and 200 µm in thickness. The endothelial cell sheets were peeled with Descemet membrane from the stromal layer. The limbal explants, stromal explants, and endothelial cell sheets were rinsed three times with Dulbecco’s phosphate buffered saline (D-PBS; with 100 IU/ml penicillin and 25µg/ml gentamicin; Sigma). Then, they were treated with 1.2 U/ml dispase II (Boehringer Mannheim) for 30 min at 37 °C in an incubator with a humidified atmosphere of 5% CO_2_. The three specimens were washed three times with Dulbecco’s modified Eagle’s medium (DMEM; with 10% fetal bovine serum) and placed directly onto 35 mm culture dishes. The side of the limbal explant epithelial cells and endothelial cell sheets were forward up and dried naturally for 15 min on a clean bench to adhere to the culture dishes. A 10% DMEM culture medium was added and was changed every three days. The corneal epithelial cells, keratocytes, and endothelial cells were culturally adhered to the culture dishes. To make single cell suspension, these were then treated with 0.25% trypsin-EDTA (Gibco-BRL) in an incubator for 10 min at 37 °C and a humidified atmosphere of 5% CO_2_. These three different cell types were directly seeded onto the epithelial, stromal, and endothelial sides of the ACMPs at 1×10^4^ cells/cm^2^ cell density. Submerged culture was two weeks, and airlifting culture was two weeks. The culture medium was changed every three days. The composition culture media was as follows: 3:1 DMEM/F12, 10% fetal bovine serum (Gibco-BRL), 1% penicillin-streptomycin, 10 ng/ml epithelial growth factor (EGF; Gibco-BRL), 5 µg/ml insulin (Gibco-BRL), 10 nM cholera toxin (Sigma), 5 µg/ml transferrin (Sigma), 4 ng/ml hydrocortisone (Sigma), and 2×10^−14^ M 3,3′,5-triiodo-L-thyronine (T3; Sigma) [22,23].

#### Colony formation efficiency (CFE)

For the cell growth assay, primary cultured rabbit corneal epithelial cells, keratocytes, and endothelial cells were passed and seeded at a density of 300 cells per dish onto 35 mm culture dishes (Corning, Glendale, AZ). Cells were cultured with the culture medium at 37 °C in an incubator with a humidified atmosphere of 5% CO_2_. The culture medium was changed every three days. Colonies were identified under an inverted microscope. After 10 days, the cells were washed three times with D-PBS and were fixed with 10% formalin for 30 min at 4 °C. The cells were stained in 2% rhodamine blue (Merck, Haar, Germany) and 2% Nile blue (Merck) mixed solution (ratio 1:1) for 30 min at 4 °C. The formed colonies were counted, and the colony formation efficiency (CFE) was identified according to following equation [24]:

CFE (%)=colony number/seeding cell number×100%

#### BrdU (5-bromo-2′-deoxyuridine) incorporation and proliferation assay

The primary cultured rabbit corneal epithelial cells, keratocytes, and endothelial cells were passed and seeded at a density of 300 cells per dish onto 15 mm culture plates (Corning). Cells were cultured at 37 °C in an incubator with a humidified atmosphere of 5% CO_2_ for 30 min with 10% FBS DMEM culture medium containing 10 µm BrdU (Chemicon, Temecula, CA). After 30 min of incubation, the medium was changed to a BrdU free medium and cultured for 24 h. The cells were fixed in 70% ethanol for 30 min at 4 °C. The fixed cells were then treated for 30 min with an anti-BrdU monoclonal antibody (Chemicon). BrdU was detected by a FITC-conjugated secondary antibody (Chemicon). The BrdU positive cells were detected using a confocal laser microscope (Zeiss, Oberkochen, Germany) [25,26].

### Biocompatibility tests in vivo and in vitro

The New Zealand white rabbits, weighing approximately 2–3 kg, were studied following the guidelines established in the ARVO statement for the use of animals in ophthalmic and vision research. All rabbits were anesthetized by intramuscular injection with xylazine hydrochloride (10 mg/kg; Bayer, Munich, Germany) and ketamine hydrochloride (60 mg/kg; Huawei). A lamellar stromal pocket (size 6×6 mm) was performed in the center of the right eye cornea. The ACMP was rinsed three times with PBS and was approximately 4×4 mm. The ACMP was inserted into the rabbit corneal stromal pocket and sutured using separate radial sutures of 10–0 nylon. After transplantation, the rabbits were examined daily with a penlight to check for graft clarity and signs of wound dehiscence, infection, or inflammation. After one, two, three, and four weeks each, each transplanted eye was examined using slit-lamp microscopy and photographed for changes in transparency and neovascularization. At specific time points the rabbit corneas were harvested under anesthesia. The primary cultured rabbit corneal limbal epithelial cells, keratocytes, and endothelial cells were passaged and seeded onto the epithelial side, keratocyte side, and endothelial side of the ACMP at a cell density of 5.0×10^3^ cells/mm^2^. After waiting 30 min for the seeded cells to adhere to the ACMP on each side, the culture medium was added and was cultured at 37 °C in a humidified atmosphere of 5% CO_2_ for four weeks. The culture medium was changed every three days [27-29].

### Scanning electron microscopy

For scanning electron microscopy (SEM), the ACMP and specimens of the three types of cell seeded ACMP were fixed with 2% glutaraldehyde in 0.1 M PBS (pH 7.2) at 37 °C for 1 h, washed three times in buffer for 10 min, fixed in 1% osmium tetroxide for 1 h at 37 °C, and dehydrated through a graded series of ethanol. Three types of fixed cell seeded ACMP specimens were critical point dried (HCP-2; Hitachi, Ibaraki, Japan), coated with platinum using an Ion Coater (IB-5; EIKO, Ibaraki, Japan), and observed on a scanning electron microscopy (Hitachi S-800; Hitachi) at magnifications between 500X and 2000X [30].

### Immunofluorescence

Immunofluorescence staining was used to detect keratin 3, vimentin, and aquaporin A for the cultured rabbit corneal epithelial cells, keratocytes, and endothelial cells. The three types of passaged rabbit corneal cells were fixed in ice-cold methanol on a culture plate for 10 min and washed in PBS. The ACMP and cell-ACMP scaffold specimens were freshly frozen in an optimal cutting temperature (OCT; Sakura Finetek, Torrance, CA) compound and then sliced with a cryostat. Section tissues were air dried. The sections of the three types of cultured cells were blocked with 2% BSA in PBS, and primary antibodies and 1:100 diluted fluorescein conjugated-goat and anti-mouse IgG secondary antibodies were applied overnight in a moist chamber set at 4 °C. The primary antibodies were anti-keratin 3 (1:100; Chemicon), anti-vimentin (1:100; Sigma), and anti-aquaporin A (1:100; Chemicon). The negative controls were prepared by incubation with the secondary antibody alone. All cells and tissues were observed on a confocal laser microscope (LSM 510; Zeiss) [31,32].

### Protein isolation and western blot analysis

The three passaged cell types were cultured to adhere to the culture plate and harvested for western blot analysis. The three harvested type cells were sonicated for 15 s intervals at 100 W. Cell debris was cleared from the lysates by centrifugation at 200x g for 15 min at 4 °C. Lysate fractions were separated into soluble and particulate using centrifugation at 29,000x g for 30 min at 4 °C. In some experiments, the cells were fractionated by low-speed centrifugation at 1000x g and high-speed centrifugation at 1,000,000x g for 15 min at 4 °C. The pellets were resuspended in modified radio immunoprecipitation assay (RIPA) buffer (50 mM Tris-HCI, pH 7.4), 1% Nonidet p-40, 0.25% sodium deoxycholate, 150 mM sodium chloride, 1 mM EDTA, 1 mM phenylmethanesulfonyl fluoride (PMSF), 5 µg/ml aprotinin, 5 µg/ml leupeptin, 1 µg/ml pepstatin, 1 mM Na_3_VO_4_, and 1 mM sodium fluoride. Protein concentrations were determined with the bicinchoninic (BCA) protein assay reagent kit (Pierce Biotechnology, Rockford, IL). Both the supernatant and particulate fractions were stored at −80 °C. Sodium dodecyl sulfate-PAGE (SDS–PAGE) was performed on Bis-Tris 4%-12% gels (NuPAGE; Novex, San Diego, CA) with MES (2-N-morpholino-ethanesulfonic acid) running buffer (Invitrogen, Carlsbad, CA). Proteins were detected on the immunoblots by chemiluminescence (SuperSignal Femto West Chemiluminescent Substrate Kit; Pierce) according to the manufacturer’s directions. Blots were developed on an autoradiograph film (CL-Xposure; Pierce and GBX developer and fixer solutions, Eastman Kodak, Rochester). Blots were reused after the antibodies, and substrates were removed with the western blot stripping buffer (Restore; Pierce) according to the manufacturer’s directions. Antibodies used in these studies included anti-keratin 3, anti-vimentin, and anti-aquaporin A [32].

### Statistics

Statistical analyses were performed using SPSS for Window, Version 11.0 (SPSS Inc., Chicago, IL). Statistical significance was set at p<0.05.

## Results

### Physical and mechanical characterization of porcine acellular corneal matrix

**Figure 1:**
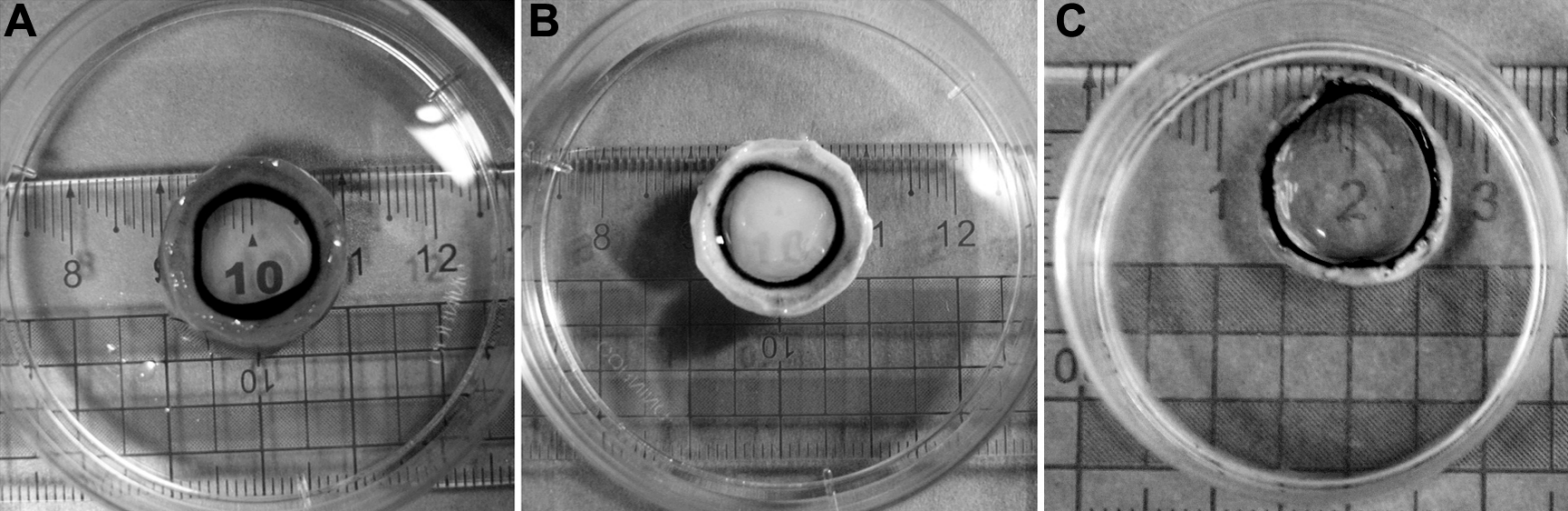
Optical properties of the ACMP. The visually opaque initial ACMP (**A**) was dehydrated by 90% glycerol for 2 h to obtain a transparency similar to a normal porcine cornea. The initial ACMP was visually opaque (**B**). Normal porcine cornea was transparent (**C**).

Visually, the initial ACMP was opaque. After dehydration, the ACMP cleared with a transparency similar to a normal porcine cornea (Figure 1). The properties of the rate of strength, expansion, water content, and light transparency of the ACMP and normal porcine cornea are shown on Table 1.

**Table 1:** Physical and mechanical characterization of porcine acellular corneal matrix.

**Contents**	**ACMP (Initial)**	**ACMP (Dehydrated 2 h)**	**ACMP (Dehydrated 4 h)**	**Normal porcine cornea**
Strength	3.07±0.62	3.34±0.84	3.39±0.23	3.51±0.64
Expansion	88.57±1.24	75.25±0.93	71.87±1.31	73.01±0.54
Ratio of water content	89.35±0.61	74.62±0.54	65.17±0.21	71.93±0.47
Ratio of light transparency	1.8±0.06	44.11±0.37	48.66±0.28	54.77±0.43

Comparison in terms of strength, expansion, and ratio of water content in initial ACMP, dehydrated ACMP and normal porcine cornea, showed no statistical significance of the differences (p>0.05) while the ratio of light transparency of initial ACMP compared to other groups showed a statistical significance of the difference (p<0.05). Statistical significance was assigned when the p value was less 0.05.

### Histological characterization of acellular corneal matrix of porcine

**Figure 2:**
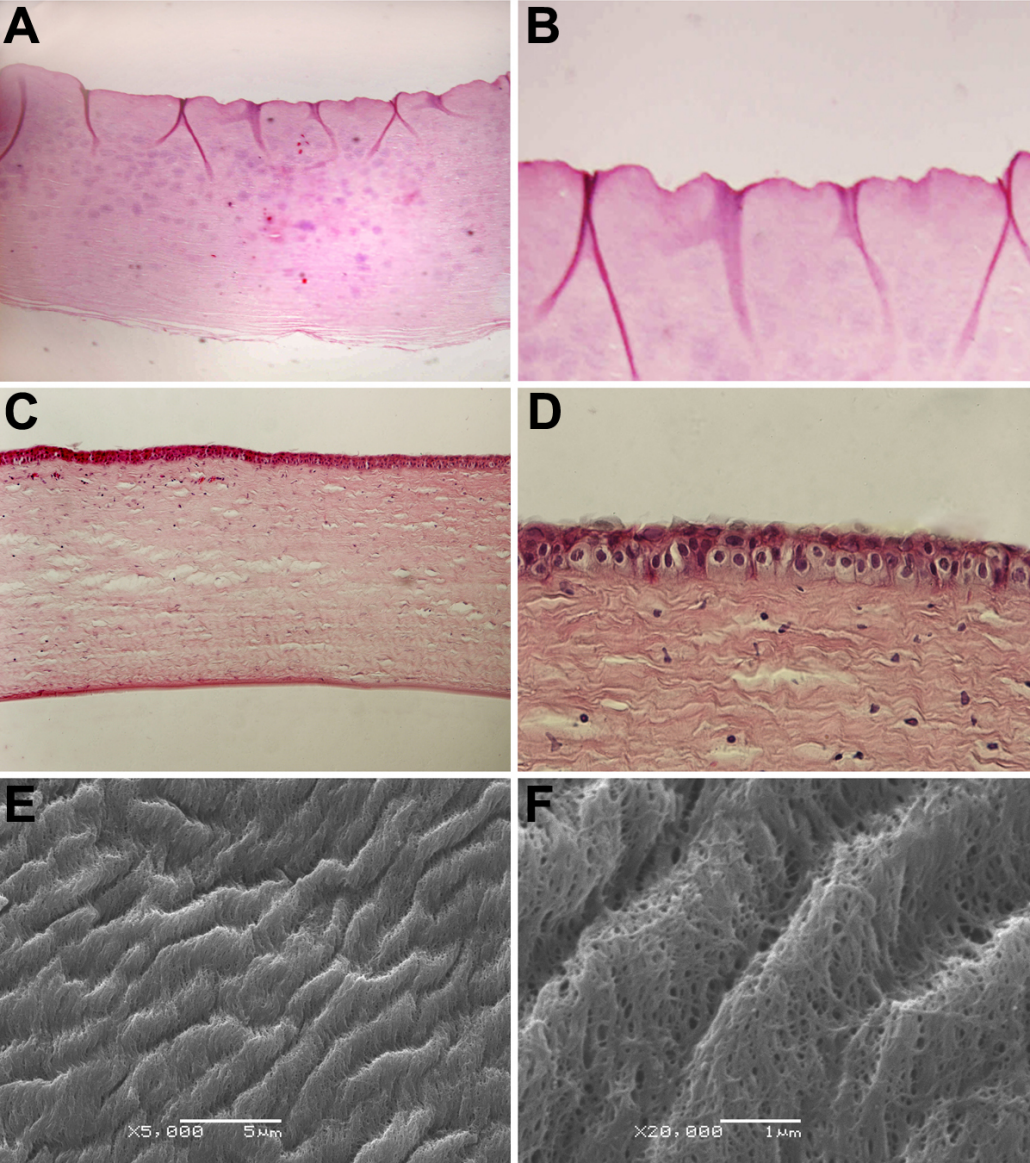
Histological characteristics of the ACMP. HE stained histological sections showed that the major immunogenic porcine corneal epithelial cells, keratocytes, and endothelial cells were completely removed in (**A**; 10X) and (**B**; 40X). The epithelial cells, keratocytes, and endothelial cells would be detected on each layer of a normal porcine cornea (**C**; 10X) and (**D**; 40X). SEM images of the ACMP showed that the collagen fibers and fibers inter-connecting to network had formed collagen bundles, which were regular and parallel to the corneal surface (**E**; 5,000X) and (**F**; 20,000X). These were similar to the normal porcine cornea matrix.

The histological characteristics of the ACMP were shown from the hematoxylin and eosin (HE) stain and scanning electron microscopy. The major immunogenic porcine corneal epithelial cells, keratocytes, and endothelial cells were completely removed by a series of extraction procedures, and the matrix architecture was well preserved compared with that of the normal porcine cornea (Figure 2A-D). Figure 2E,F show that the collagen fiber diameter and the fiber interconnected to the network formed collagen bundles both regular and parallel to the corneal surface. These were similar to the normal porcine cornea matrix.

### Biocompatibility tests of porcine acellular corneal matrix

#### Implantation and clinical evaluation

**Figure 3:**
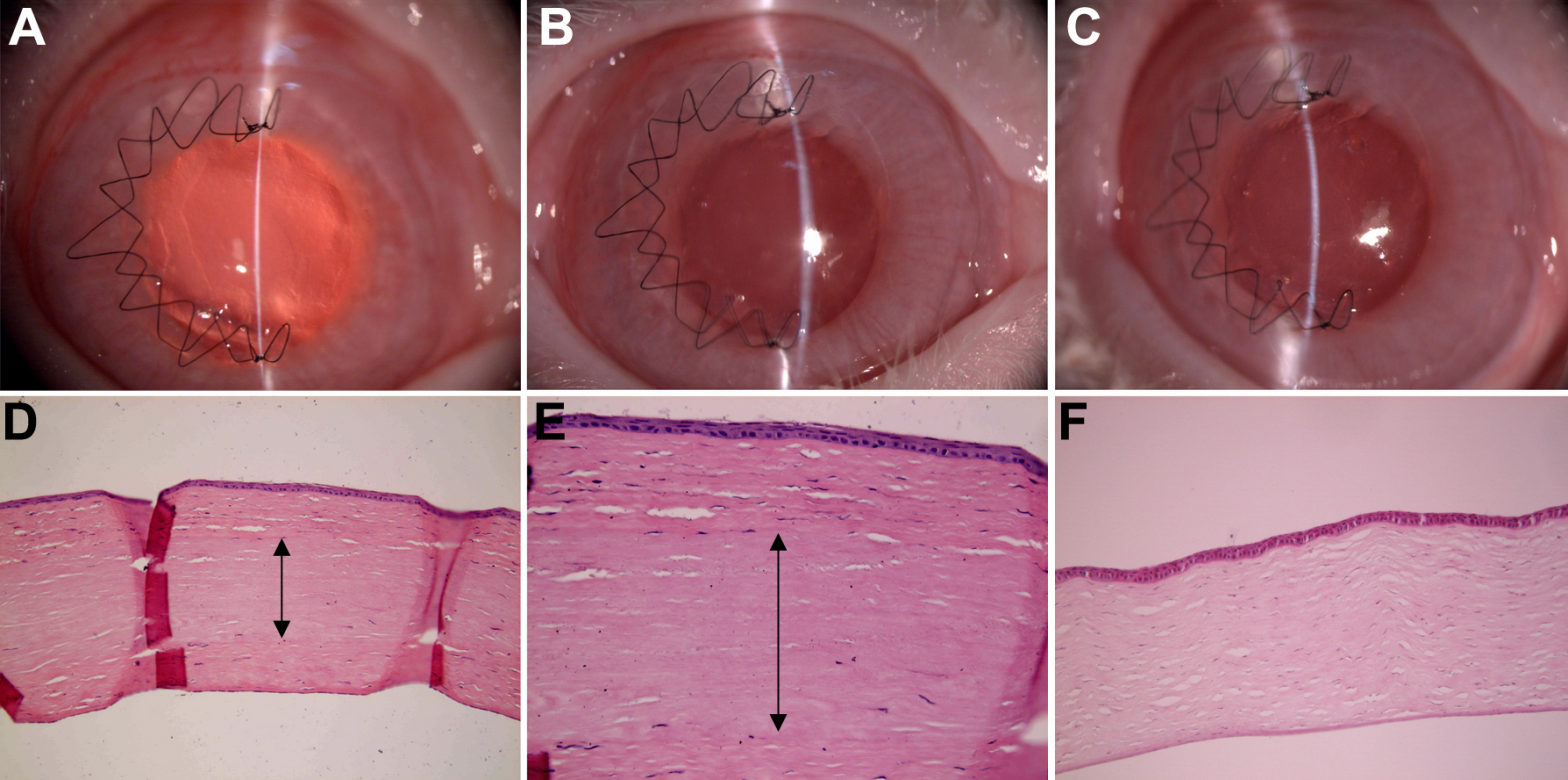
Investigate the biocompatibility of ACMP for rabbit cornea. The ACMP was inserted into a rabbit corneal stromal pocket and observed over one month of post surgical period. The cornea showed a mild haze at one day (**A**), optically clear slit lamp images at one week (**B**) and one month (**C**) and no inflammation or rejection signs in (**D**; 10X) and (**E;** 20X). The normal cornea is presented in (**F**) as a control (10X).

Slit lamp examination was performed one, two, three, and four weeks after surgery on eight rabbit right eyes and showed reepithelialization within the first week. Although a mild haze was initially observed, none of the ACMP implants showed any inflammation or rejection signs over this period. In the rabbit series, the epithelium was beginning to heal one week after surgery (Figure 3B). One month after surgery, rabbit cornea sections showed a stratified epithelium, and stromal cells had migrated into the implant region according to HE staining (Figure 3D,E). Compared to the normal rabbit cornea (Figure 3F), the ACMP implanted rabbit cornea showed a similar normal rabbit stromal histological appearance, and the implants were well integrated within the host after one month of surgery. None of the implants showed any inflammation cells or neovascular invasion in the corneas over this period.

#### In vitro performance

**Figure 4:**
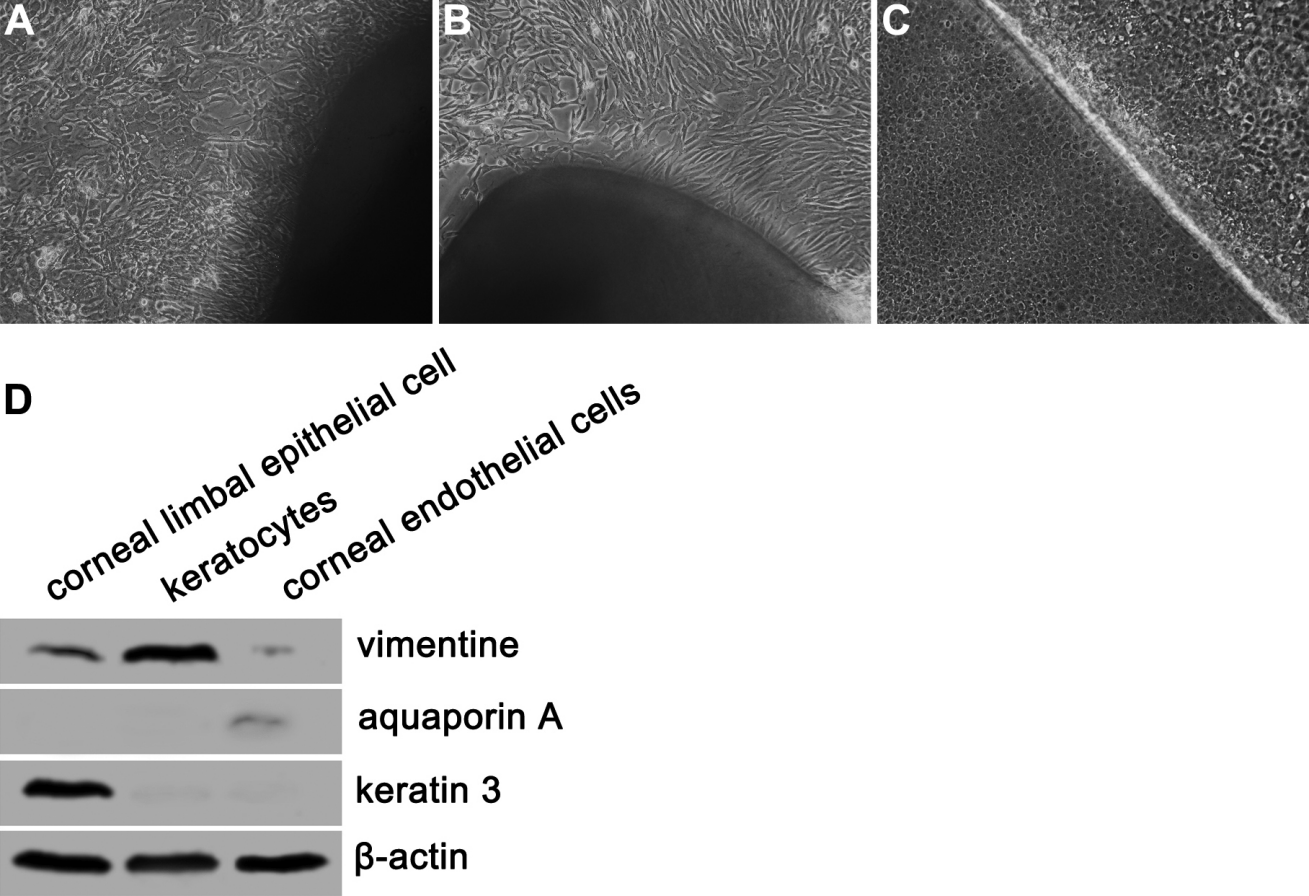
Phase appearance and marker protein expression of cultivated corneal limbal epithelial cells, keratocytes, and endothelial cells. The rabbit corneal limbal epithelial cells (**A**), keratocytes (**B**), and endothelial cells (**C)** were cultivated for one week to confluence on culture dishes, primary (10X). The protein of keratin 3 was specially expressed in the cultivated corneal limbal epithelial cells while vimentin expression was highest in the keratocytes. Aquaporin A was only detected in the endothelium (**D**).

**Figure 5:**
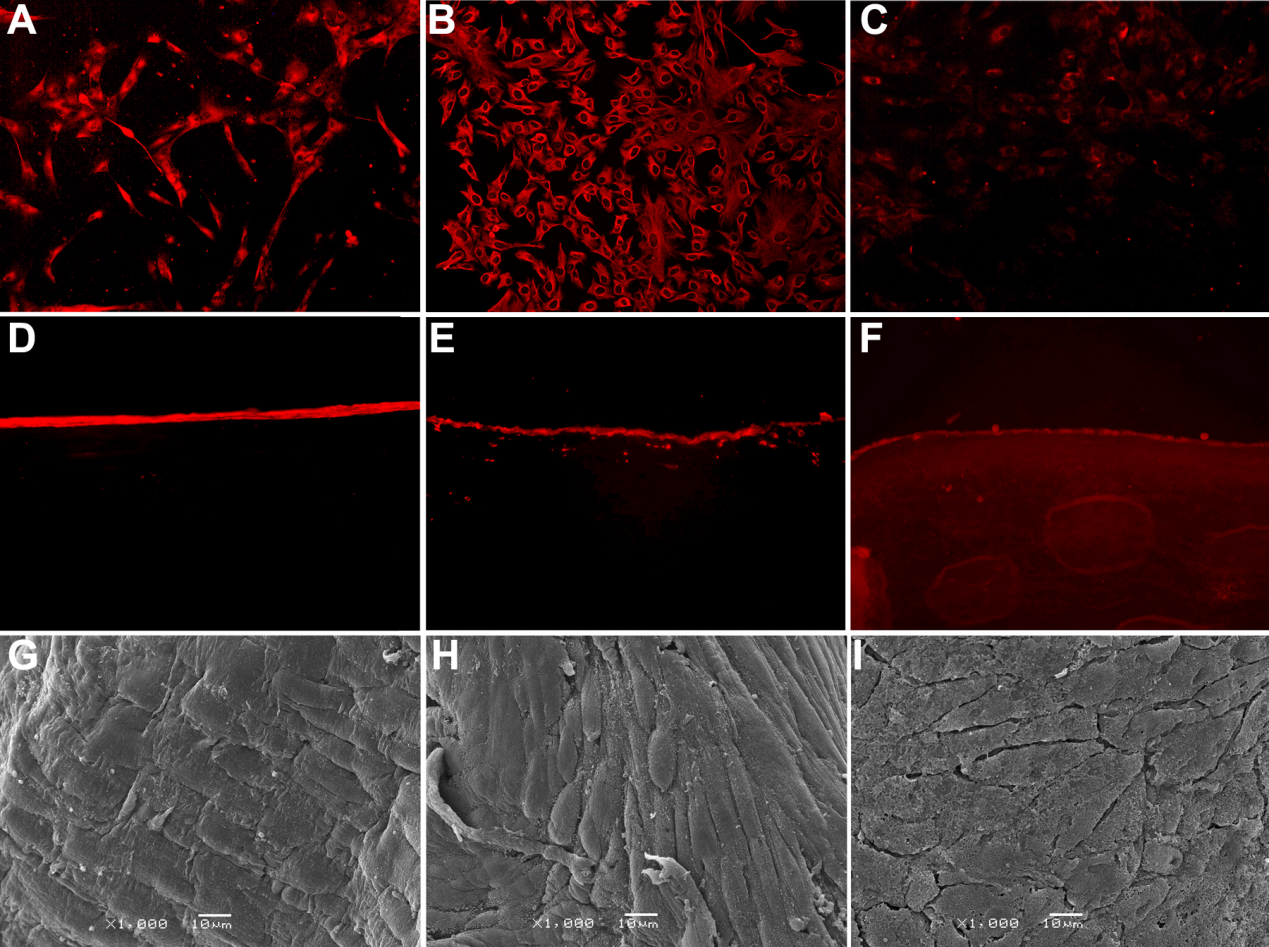
Rabbit corneal epithelium-ACMP, keratocyte-ACMP, and endothelium-ACMP scaffold was built in vitro. The cultivated rabbit corneal epithelial cells, keratocytes, and endothelial cells were positive for keratin 3, vimentin, and aquaporin A following immunofluorescence staining (**A**, **B**, and **C**, respectively) and these three types of cultivated cells were seeded onto ACMP. The epithelium-ACMP, keratocyte-ACMP, and endothelium-ACMP scaffold were built, respectively (**D**, **E**, and **F**). SEM showed that these three types of cultivated cells adhered to the surface of ACMP, and maintained their conformation (**G**, **H**, and **I**).

To examine the compatibility of the ACMP with the cultured rabbit corneal limbal epithelial cells, keratocytes, and endothelial cells, the three rabbit corneal cell types were separated from the special corneal tissues and were cultured in vitro. As Figure 4A-C shows, three cell types were moved from the tissues and were grown on the culture plates. The proliferations of these three cell types were tested by colony formation efficiency and BrdU proliferation assay. After 10 days in culture, the three cell types had good growth and formed large colonies. The colony formation efficiency was 23.16±3.61% for limbal epithelial cells, 18.16±3.12% for keratocytes, and 11.72±1.42% for endothelial cells (p<0.05). Counting the continuously cultured corneal limbal epithelial cells, keratocytes, and endothelial cells, which one set was stained with BrdU for three days and another stained for seven days, the BrdU staining indexes were 13.8%, 21.3%, and 17.2% after three days and 42.2%, 51.5%, and 48.1% after seven days, respectively (p<0.05). The expressions of the marker proteins were examined by western blotting (Figure 4D). Keratin 3 protein was expressed in the cultured corneal limbal epithelial cells while vimentin expression was highest in the keratocytes. Aquaporin A was only detected in the slender band in the endothelium, and the bands were thick. The passaged rabbit corneal limbal epithelial cells, keratocytes, and endothelial cells formed a confluent cell sheet on the ACMP, consisting of one or two cell layers. No changes in cell properties or in morphological characteristics were observed. The three cell types maintained their shape and specific molecule expression in cell-ACMP scaffolds such as keratin 3, vimentin, and aquaporin A (Figure 5D-F), which was the same as the three cultured cell types (Figure 5A-C). SEM examination results showed that the cells steadily adhered to the surface of the ACMP and maintained their conformation (Figure 5G-H).

## Discussion

The replacement of diseased tissues and organs with bioengineered tissue is rapidly moving from the realm of science fiction to reality. Elegant and innovative work in laboratories around the world has demonstrated the feasibility of similar approaches for bioengineering corneal replacements [2,3,6]. Successful tissue engineering is known to depend on the provision of a promising scaffold during the initial stages of reconstruction [33,34]. This is necessary so that the cells in an implant can proliferate, move out onto a framework, and lay down extracellular matrix. At present, synthetic and natural biological materials are used to provide scaffolding support for tissue engineering, and compared with synthetic biomaterials, natural biomaterials are more promising because of their flexibility and suitable physical and mechanical properties. Moreover, many biomaterials demonstrate physiologic and biochemical functions equivalent to the normal cornea, but each has a limitation preventing its current use in humans [3,14,35]. Many corneal wounds and scarring diseases persist for weeks and months or else recur frequently and even progress to corneal perforation. Our major aim in developing an artificial epithelium scaffold, stroma scaffold, and endothelium scaffold by tissue engineering is to provide a cure for those individuals afflicted with severe corneal wounds associated with a deeper involvement of the epithelial basement membrane and of stromal lamella or endothelium [20,36].

Multilayered cultured corneal cells can be generated by using tissue culture techniques, and these limbal epithelial, keratocytes, and endothelial cell sheets have been successfully grafted to damaged eyes. However, the fragility of these grafts by lacking a scaffold makes them difficult to handle and subject to easy shearing from the recipient eye. Thus, analogue of engineering tissue is to contain a compatible scaffold component. The amniotic membrane is the logical choice as a source of the biomaterials in corneal transplantation [8,11,37]. However, its use greatly limits the characteristics of the membrane. Our approach in tissue engineering the corneal epithelial scaffold, stroma scaffold, and endothelial scaffold is based on the porcine acellular matrix. This is intuitively attractive because of its anatomic similarity to the normal corneal stroma, and biocompatibility tests have shown that the ACMP is an ideal biomaterial because limbal epithelial cells, keratocytes, and endothelial cells can fill the surface and produce extracellular matrix. In this study, we have tested the efficiency of a tissue engineered ACMP. Our findings demonstrate that ACMP is a suitable biocompatible material on which epithelial cells, keratocytes, and endothelial cells may attach and proliferate in vitro. Although our experiment is not adequate for clinical use on humans [38,39], proper biocompatibility and the basic requirement for the tissue engineered cornea has subsequently been confirmed by histology. The ACMP gradually degrade and become absorbed, accompanied by an in growth of new tissue. The tissue engineering of corneal scaffolds results in the fabrication of a useful corneal lamella equivalent with respect to gross appearance, optical clarity, and histological structure.

The maintenance of corneal transparency is dependent upon the integrity of its three distinct layers, the epithelium, stroma, and endothelium. All these layers are essential because they elicit the outward transport of net ions from the stroma and provide the driving force for fluid transport into tears and the anterior chamber. In detail, a major function of the corneal endothelium is to maintain corneal transparency by regulating corneal hydration. Proteoglycans associated with stromal collagens bind water and produce a pressure gradient across the endothelium. This is indicated by our finding that tissue engineered construct transplantation does not cause the cornea to become translucent immediately. This is because transparency of the cornea is maintained by tight junctions in the epithelium that control the flow of fluid into the eye. Intact epithelial and endothelial cells of the regenerative corneal tissue suggest that our corneal product is likely help to treat some corneal diseases. Furthermore, the cornea is one of the most densely innervated tissues in the body and is richly supplied by sensory and autonomic nerve fibers. Nerve bundles enter the cornea at the periphery in a radial fashion parallel to the corneal surface [6,9,10,40].

Therefore, in our experiments, tissue engineered corneal epithelial, stromal, and endothelial scaffolds may act as a physiologically functional tissue substitute and not simply as a prosthetic device. Tissue engineered tissue replacements may represent the future for the repair of many tissues and organs. For the cornea, we are closer to the realization of this prospect than for other tissues. Our study demonstrates that the tissue engineered corneal epithelial, stromal, and endothelial scaffolds were constructed with ACMP and cultured limbal epithelial cells, keratocytes, and endothelial cells in vitro [41,42]. In summary, transplantations with three types of tissue engineered corneal scaffolds may be an effective and safe treatment to facilitate the rapid restoration of the cornea without causing complications. Although progress has been achieved, tissue engineered corneal replacements can be easily produced and transplanted. Our laboratory and many others will continue to pursue this goal of readily available corneal replacements.

## References

[1] Zhao B Cooper LJ Brahma A MacNeil SRimmer S, Fullwood NJDevelopment of a three-dimensional organ culture model for corneal wound healing and corneal transplantation. Invest Ophthalmol Vis Sci 2006 47 2840 5 16799023 10.1167/iovs.05-1367

[2] Usas A, Huard J (2007). Muscle-derived stem cells for tissue engineering and regenerative-therapy.. Biomaterials.

[3] Kampmeier J, Radt B, Birngruber R, Brinkmann R (2000). Thermal and biomechanical parameters of porcine cornea.. Cornea.

[4] Bell E, Ehrlich HP, Buttle DJ, Nakatsuji T (1981). Living tissue formed in vitro and accepted as skin-equivalent tissue of full thickness.. Science.

[5] Chen J Wang C Lu S Wu J Guo X Duan C Dong L Song Y Zhang JJing D, Wu L, Ding J, Li DIn vivo chondrogenesis of adult bone-marrow-derived autologous mesenchymal stem cells. Cell Tissue Res 2005 319 429 38 15672263 10.1007/s00441-004-1025-0

[6] Fan XQ, Chen P, Fu Y (2007). Xenogenic corneal acellular matrix as carrier for reconstruction of biological cornea epithelium-scaffold-endothelium compound.. Zhonghua Yan Ke Za Zhi.

[7] Griffith LG, Naughton G (2002). Tissue engineering: current challenges and expanding opportunities.. Science.

[8] Griffith M, Osborne R, Munger R, Xiong X, Doillon CJ, Laycock NL, Hakim M, Song Y, Watsky MA (1999). Functional human corneal equivalents constructed from cell lines. Science.

[9] Heinz C, Eckstein A, Steuhl KP, Meller D (2004). Amniotic membrane transplantation for reconstruction of corneal ulcer in Graves ophthalmopathy.. Cornea.

[10] Hu X, Lui W, Cui L, Wang M, Cao Y (2005). Tissue engineering of nearly transparent corneal stroma.. Tissue Eng.

[11] Ignacio TS, Nguyen TT, Sarayba MA, Sweet PM, Piovanetti O, Chuck RS, Behrens A (2005). A technique to harvest decrement’s membrane with viable endothelial cells for selective transplantation.. Am J Ophthalmol.

[12] Engelmann K, Bednarz J, Schafer HJ, Friedl P (2001). Isolation and characterization of a mouse monoclonal antibody against human corneal endothelial cells.. Exp Eye Res.

[13] Koizumi N, Inatomi T, Suzuki T, Sotozono C, Kinoshita S (2001). Cultivated corneal epithelial stem cell transplantation in ocular surface disorders.. Ophthalmology.

[14] Meller D, Dabul V, Tseng SC (2002). Expansion of conjunctival epithelial progenitor cells on amniotic membrane.. Exp Eye Res.

[15] Meller D, Pires RT, Tseng SC (2002). Ex vivo preservation and expansion of human limbal epithelial stem cells on amniotic membrane cultures.. Br J Ophthalmol.

[16] Minami Y, Sugihara H, Oono S (1993). Reconstruction of cornea in three-dimensional collagen gel matrix culture.. Invest Ophthalmol Vis Sci.

[17] L’Heureux N, Dusserre N, Konig G, Victor B, Keire P, Wight TN, Chronos NA, Kyles AE, Gregory CR, Hoyt G, Robbins RC, McAllister TN (2006). Human tissue- engineered blood vessels for adult arterial revascularization.. Nat Med.

[18] Orwin EJ, Hubel A (2000). In vitro culture characteristics of corneal epithelial, endothelial and keratocyte cell in a native collagen matrix.. Tissue Eng.

[19] Reichl S, Bednarz J, Muller-Goymann CC (2004). Human corneal equivalent as cell culture model for in vitro drug permeation studies.. Br J Ophthalmol.

[20] Sarasam A, Madihally SV (2005). Characterization of chitosan-polycaprolactone blends for tissue engineering applications.. Biomaterials.

[21] Schwab IR, Reyes M, Isseroff RR (2000). Successful transplantation of bioengineered tissue replacements in patients with ocular surface disease.. Cornea.

[22] Tsai RJ, Li LM, Chen JK (2000). Reconstruction of damaged corneas by transplantation of autologous limbal epithelial cells.. N Engl J Med.

[23] Zhang C, Jin Y, Nie X, Liu Y, Lei J, Hu D (2006). A comparative study on biocompatibility of acellular corneal stroma materials prepared by serial digestion methods.. Zhongguo Xiu Fu Chong Jian Wai Ke Za Zhi..

[24] Wilson SE, Netto M, Ambrosio R (2003). Corneal cells:chatty in development, homeostasis, wound healing, and disease.. Am J Ophthalmol.

[25] Wilson SE, Liu JJ, Mohan RR (1999). Stromal-epithelial interactions in the cornea.. Prog Retin Eye Res.

[26] Hu X, Lui W, Cui L, Wang M, Cao Y (2005). Tissue engineering of nearly transparent corneal stroma.. Tissue Eng.

[27] Trinkaus-Randall V, Leibowitz HM, Ryan WJ, Kupferman A (1991). Quantification of stromal destruction in the inflamed cornea.. Invest Ophthalmol Vis Sci.

[28] Quantock AJ, Sano Y, Young RD, Kinoshita S (2005). Stromal architecture and immune tolerance in additive corneal xenografts in rodents.. Acta Ophthalmol Scand.

[29] Pellegrini G, Traverso CE, Franzi AT, Zingirian M, Cancedda R, De Luca M (1997). Long-term restoration of damaged corneal surfaces with autologous cultivated corneal epithelium.. Lancet.

[30] Orwin EJ, Hubel A (2000). In vitro culture characteristics of corneal epithelial, endothelial and keratocyte cells in a native collagen matrix.. Tissue Eng.

[31] Ohno K, Nelson LR, Mitooka K, Bourne WM (2002). Transplantation of cryopreserved human corneas in a xenograft model.. Cryobiology.

[32] Nakazawa K, Takahashi I, Ohno Y, Sato M (1997). Modification of proteoglycan synthesis by corneal stromal cells on co-culture with either epithelial or endothelial cells.. J Biochem.

[33] Mohan RR, Hutcheon AE, Choi R, Hong J, Lee J, Mohan RR, Ambrosio R, Zieske JD, Wilson SE (2003). Apoptosis, necrosis, prokiferation and myofibroblast generation in the stroma following LASIK and PRK.. Exp Eye Res.

[34] Kim JS, Kim JC, Na BK, Jeong JM, Song CY (2000). Amniotic membrane patching promotes healing and inhibits proteinase activity on wound healing following acute corneal alkali burn.. Exp Eye Res.

[35] Kenyon KR, Tseng SC (1989). Limbal autograft transplantation for ocular surface disorders.. Ophthalmology.

[36] Larkin DF, Calder VL, Lightman SL (1997). Identification and characterization of cells infiltrating the graft and aqueous humour in rat corneal allograft rejection.. Clin Exp Immunol.

[37] O’Brien WJ, Krema C, Heimann T, Zhao H (2006). Expression of NADPH Oxidase in rabbit corneal epithelial and stromal cells in culture.. Invest Ophthalmol Vis Sci.

[38] Oksuz H, Duran N, Tamer C, Cetin M, Silici S (2005). Effect of propolis in the treatment of experimental. Staphylococcus aureus keratitis in rabbits.. Ophthalmic Res.

[39] Ullrich V, Bachschmid M (2000). Superoxide as a messenger of endothelial function.. Biochem Biophys Res Commun.

[40] Mimura T, Yamagami S, Yokoo S, Usui T, Tanaka K, Hattori S, Irie S, Miyata K, Araie M, Amano S (2004). Cultured human corneal endothelial cell transplantation with a collagen sheet in a rabbit model.. Invest Ophthalmol Vis Sci.

[41] Miyata K, Drake J, Osakabe Y, Hosokawa Y, Hwang D, Soya K, Oshika T, Amano S (2001). Effect of donor age on morphologic variation of cultured human corneal endothelial cells.. Cornea.

[42] Bednarz J, Weich HA, Rodokanaki-von Schrenck A, Engelmann K (1995). Expression of genes coding growth factors and growth factor receptors in differentiated and dedifferentiated human corneal endothelial cells.. Cornea.

